# Pathogen surveillance and risk factors for pulmonary infection in patients with lung cancer: A retrospective single-center study

**DOI:** 10.1515/med-2025-1180

**Published:** 2025-04-30

**Authors:** Hu Shan, Jing Wang, Qiuhong Zhang, Zongjuan Ming, Yonghong Zhang, Ping He, Ping Fang, Ming Zhang, Wei Li, Hongyang Shi, Yuanlin Guan, Shuanying Yang

**Affiliations:** Department of Respiratory and Critical Care Medicine, The Second Affiliated Hospital of Xi’an Jiaotong University, Shaanxi, China; Department of Scientific Affairs, Hugobiotech Co., Ltd., Beijing, China

**Keywords:** pulmonary infection, lung cancer, pathogens, co-infection, mNGS

## Abstract

**Background:**

Early and accurate diagnosis of pulmonary infection (PI) is crucial for the timely implementation of appropriate treatment strategies in lung cancer patients.

**Methods:**

Metagenomic next-generation sequencing and conventional testing were performed in lung cancer patients with and without PI. The pathogen profiles were analyzed, and risk factors for PI were explored using univariate and multivariate logistic regression models.

**Results:**

A total of 55 lung cancer patients with PI and 59 non-infected lung cancer patients were included. There were 41 underlying pathogens identified by both methods in lung cancer patients with PI. The coexistence of different pathogen types was common, particularly between fungi and viruses, which was observed in 28.57% of cases. The incidence of *Streptococcus pneumoniae* and *Pneumocystis jirovecii* is significantly higher in small-cell lung carcinoma patients compared to that in non-small-cell lung carcinoma patients. Besides, cytomegalovirus, *P. jirovecii*, and *Aspergillus* were more likely to be found in advanced-stage patients. Risk factor analysis revealed that Karnofsky Performance Status <90 and chemotherapy were strongly associated with PI in lung cancer patients.

**Conclusions:**

This study highlights the complexity of PI in lung cancer patients, emphasizing the need for tailored diagnostic and therapeutic strategies based on cancer type and stage.

## 1 Introduction

Lung cancer remains a significant global public health challenge, characterized by its high incidence and mortality rates [1]. In China, the 2022 cancer statistics reported approximately 0.87 million new cases and 0.77 million deaths due to lung cancer [2]. Among lung cancer patients, pulmonary infections (PI) present a frequent and significant issue. The predisposition to PI in these patients is driven by factors such as compromised anatomical barriers from invasive procedures and immune deficiencies associated with cancer [3,4,5]. These infections not only impede the efficacy of cancer treatments but also significantly impact overall survival, contributing substantially to lung cancer-related mortality [6,7].

Early diagnosis and timely treatment of PI are crucial for improving outcomes in lung cancer patients. Previous research has shown that a delay of more than 5 days in identifying the cause of pulmonary infiltrates can increase the risk of mortality [8]. Although data on microbial infections in lung cancer patients are limited, existing evidence highlights a predominance of gram-negative (G−) bacteria in PI within this population [9,10]. Beyond conventional or antibiotic-resistant pathogens, the increasing prevalence of opportunistic infections must also be considered in the management of PI in lung cancer patients [11].

In recent years, clinical metagenomic next-generation sequencing (mNGS) has been increasingly utilized for pathogen detection, offering several advantages over conventional methods (CTM) [12,13,14]. As an unbiased, high-throughput sequencing technology, mNGS comprehensively analyzes all nucleic acids present in a sample, enabling the simultaneous identification of a diverse array of microorganisms, including bacteria, viruses, fungi, and parasites, in a single run. Compared to traditional culture-based methods, mNGS provides faster, broader-spectrum, and highly sensitive pathogen detection, with results that are less likely to be influenced by prior antibiotic use. This makes mNGS particularly valuable for immunocompromised patients, who are at higher risk of mixed infections and opportunistic pathogens, as it can effectively detect complex and co-existing infections that might otherwise be overlooked by conventional diagnostic approaches.

In this study, we aimed to elucidate the spectrum and characteristics of pathogens causing PI in lung cancer patients by integrating the results of mNGS with CTM. Additionally, we sought to evaluate the risk factors associated with PI in this vulnerable population.

## 2 Methods

### 2.1 Study design

Inpatients diagnosed with lung cancer at the Second Affiliated Hospital of Xi’an Jiaotong University from January 2021 to August 2023 were enrolled consecutively in this study. The case group was patients with PI who underwent both mNGS and conventional diagnostic testing using bronchoalveolar lavage fluid (BALF). The diagnosis of PI was based on: (1) imaging examinations revealing lung infiltrates, increased density in the lesion area, blurred borders, cavities, and fluid accumulation; (2) at least one of the following typical clinical characteristics: (a) new-onset cough, sputum production, dyspnoea, chest pain, or exacerbation of existing respiratory symptoms, (b) fever, and (c) peripheral leukocytosis (>10 × 10^9^/L) or leucopenia (<4 × 10^9^/L); and (3) integration of mNGS results, conventional diagnostic methods, and clinical judgment by clinicians. The control subjects were consecutive non-infected patients who did not exhibit typical clinical characteristics of PI. The control group consisted of non-infected patients who did not exhibit typical clinical characteristics of PI. Controls were frequency matched to the case patients by sex, type of lung cancer, and TNM stage of lung cancer, with an approximate 1:1 ratio. The patients were staged according to the guidelines of the eighth edition of the lung cancer staging system of the American Joint Committee on Cancer [15]. Patients with incomplete clinical data, those who declined to provide informed consent, and individuals with COVID-19 infection were excluded from the study.

### 2.2 Pathogen profiles evaluation

The landscape of pathogens in lung cancer patients with PI was assessed by combining conventional laboratory tests and mNGS analysis (Hugobiotech, Beijing, China) of BALF samples [16]. The conventional laboratory tests included bacterial and fungal culture, antigen measurement, β-1,3-glucans (G) test, and galactomannan test. In addition, PCR was performed to detect 13 common respiratory pathogens (*Mycoplasma pneumoniae*, *Bordetella pertussis*, *Bordetella parapertussis*, influenza A virus, influenza B virus, parainfluenza virus, respiratory syncytial virus, adenovirus, rhinovirus, human metapneumovirus, bocavirus, coronavirus, and SARS-CoV-2). For patients suspected of tuberculosis, acid-fast staining and T-SPOT.TB were used for detection.

BALF samples were collected and sent to Hugobiotech Co., Ltd. (Beijing, China) for mNGS detection. DNA was extracted using the QIAamp DNA Micro Kit (QIAGEN, Hilden, Germany) according to its manual. DNA libraries were constructed with the QIAseq™ Ultralow Input Library Kit for Illumina (QIAGEN, Hilden, Germany). The quality and quantity of each library were measured using a Qubit fluorometer (Thermo Fisher Scientific, MA, USA) and Agilent 2100 Bioanalyzer (Agilent Technologies, Palo Alto, USA). Qualified libraries were sequenced on the Nextseq 550 platform (Illumina, San Diego, USA). After sequencing, raw data were filtered to remove adapter sequences, short reads (<35 bp), low-quality reads (*Q* < 30), and low-complexity reads. Human reads were excluded by mapping to the human reference genome (hg38). The remaining reads were aligned to Microbial Genome Databases. Negative controls (NTC, sterile deionized water) and positive controls (known quantities of synthetic fragments) were included in each batch of experiments, following the same wet lab and bioinformatics procedures. For detected bacteria (excluding *Mycobacterium tuberculosis*), fungi (excluding *Cryptococcus*), and parasites, a positive result was considered if the coverage ranked in the top 10 of similar species/genera and was absent in NTC, or if the RPM ratio between the sample and NTC (RPM_sample_/RPM_NTC_) was greater than 10 when RPM_NTC_ was not zero. For viruses, *M. tuberculosis*, and *Cryptococcus*, a positive mNGS result was indicated by the detection of at least one species-specific read and its absence in NTC, or an RPM_sample_/RPM_NTC_ ratio greater than 5 when RPM_NTC_ was not zero.

The pathogen profiles were classified as follows: identical detection of mNGS with CTM, extra detection of mNGS, false positive detection of mNGS, extra detection of CTM, and false positive detection of CTM. The false positive detection of mNGS refers to microorganisms detected only by mNGS but are diagnosed as non-causative pathogens. The false positive detection of CTM refers to microorganisms detected only by CTM but are diagnosed as non-causative pathogens.

### 2.3 Statistical analysis

Continuous variables are presented as median and interquartile ranges (IQR), and categorical variables as numbers and percentages. Continuous variables to compare the differences between infected and non-infected patients were assessed using a Wilcoxon sum test, and categorical variables were analyzed using Chi-square analysis or Fisher’s exact test as appropriate. The odds ratio (OR) and 95% confidence interval were calculated.

To explore the risk factors for PI, univariate and multivariate logistic regression models were used. Univariate logistic regression analysis was performed for some variables (relevant baseline, clinical course, and treatments) to identify parameters associated with IFIs. Multivariate analysis with logistic regression models, via a stepwise procedure, was used to identify the parameters most associated with PI. Two-sided tests were used and considered statistically significant for *P* values less than 0.05. All statistical analyses were performed with R software (version 4.1.3) with the following packages: plyr, rms, epiDisplay, and gtsummary.


**Informed consent:** Informed consent was obtained from all subjects and/or their legal guardian(s).
**Ethical approval:** This study was reviewed and approved by the Clinical Research Ethics Committee of The Second Affiliated Hospital of Xi’an Jiaotong University. All procedures were in strict compliance with the Ethical Review of Biomedical Research Involving Human Subjects (2016), the principles outlined in the Declaration of Helsinki, and the International Ethical Guidelines for Biomedical Research Involving Human Subjects.

## 3 Results

### 3.1 Baseline characteristics

In this study, a total of 114 lung cancer patients were included, comprising 55 (48.25%) patients diagnosed with PI (Case group) and 59 non-infected patients (Control group). Demographic, clinical, and tumor characteristics of the patients are shown in Table 1. The median age of the patients was 64 (IQR 58–69) years, with no significant difference observed between the case and control groups (64 [58–69.5] vs 63 [57–67.5], *P* = 0.27). Underlying diseases were present in 105 (92.11%) patients, with hypertension being the most common, followed by chronic obstructive pulmonary disease (COPD) and diabetes. Notably, the prevalence of COPD was significantly higher in patients with PI than in non-infected controls (29.09% vs 13.56%, *P* = 0.04). Besides, chemotherapy‐induced myelosuppression and non-infectious pneumonitis were found in 43.86 and 18.42% of the patients, respectively. There was no significance in other underlying diseases or comorbidities between patients with and without PI.

**Table 1:** Baseline characteristics

	Total (*n* = 114)	Patients with PI (*n* = 55)	Controls (*n* = 59)	*P* value
Male	83 (71.81%)	39 (70.91%)	44 (74.58%)	
Female	31 (27.19%)	16 (29.09%)	15 (25.42%)	
Age (median [min–max])	64 (30–82)	64 (48–82)	63 (30–79)	0.27
**Lung cancer**				
Adenocarcinoma	51 (44.74%)	21 (38.18%)	30 (50.85%)	
SqCC	41 (35.96%)	21 (38.18%)	20 (33.90%)	
Small cell carcinoma	17 (14.91%)	7 (12.73%)	10 (16.95%)	
Others	6 (5.26%)	6 (10.91%)	0	
**TNM stage**				
I	10 (8.77%)	7 (12.73%)	3 (5.08%)	
II	8 (7.02%)	6 (10.91%)	2 (3.39%)	
III	36 (31.58%)	17 (30.91%)	19 (32.20%)	
IV	60 (52.63%)	25 (45.45%)	35 (59.32%)	
**Underlying diseases/comorbidities**	105 (92.11%)	52 (94.55%)	53 (89.83%)	
Hypertension	27 (23.68%)	12 (21.82%)	15 (25.42%)	0.65
Coronary heart disease	9 (7.89%)	5 (9.09%)	4 (6.78%)	0.65
Diabetes mellitus	17 (14.91%)	9 (16.36%)	8 (13.56%)	0.67
COPD	24 (21.05%)	16 (29.09%)	8 (13.56%)	0.04
ILD	4 (3.51%)	2 (3.64%)	2 (3.39%)	0.94
Emphysema	5 (4.39%)	2 (3.64%)	3 (5.08%)	0.71
Pulmonary fibrosis	9 (7.89%)	4 (7.27%)	5 (8.47%)	0.81
Anemia	5 (4.39%)	5 (9.09%)	0	0.02
Non-infectious pneumonitis	21 (18.42%)	11 (20%)	10 (16.95%)	0.68
Chemotherapy‐induced myelosuppression	50 (43.86%)	25 (45.45%)	25 (42.37%)	0.74
Tracheoesophageal fistula	2 (1.75%)	2 (3.64%)	0	0.14

PI: pulmonary infection; TNM: tumor node metastasis; COPD: chronic obstructive pulmonary disease; ILD: interstitial lung disease.

Regarding tumor characteristics, the most common lung cancer type was lung adenocarcinoma (ADC) (51, 44.74%), followed by lung squamous cell carcinoma (SqCC) (41, 35.96%) and small cell carcinoma (17, 14.91%). The histological subtype was not defined in six patients. The majority of patients exhibited advanced-stage cancer, including 36 (31.58%) with stage III and 60 (52.63%) with stage IV. Three patients succumbed to PI after appropriate treatment, while the rest experienced favorable outcomes.

### 3.2 The landscape of pathogens identified in lung cancer patients with PI

**Figure 1:**
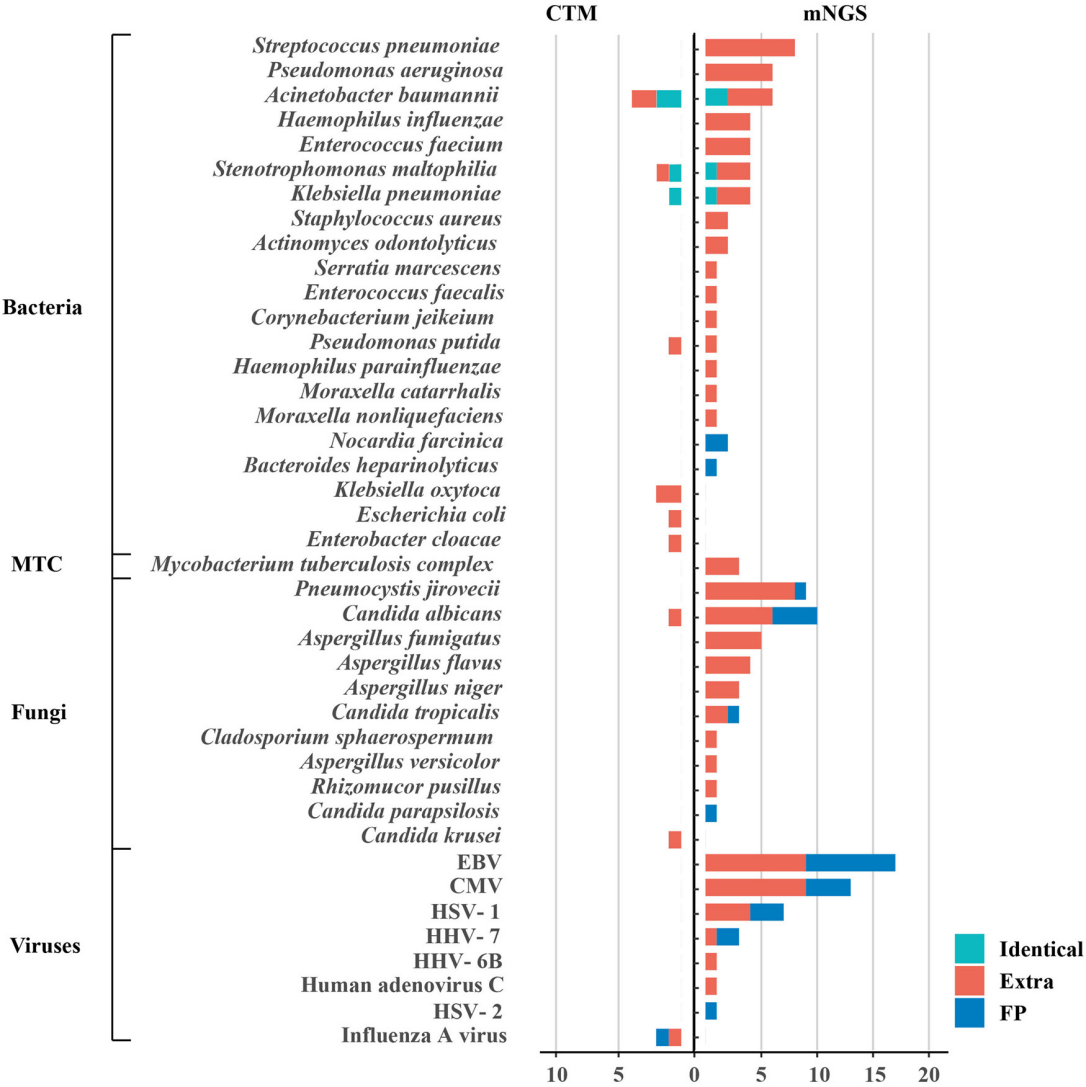
Potential pathogens detected by mNGS and CTM. Fungi and viruses were the most frequently detected pathogens, followed by G− and G+ bacteria. The bar graph shows the number of cases in which each pathogen was detected out of the 63 infection episodes. Pathogens are categorized into three groups: identical detection (green): pathogens detected by both mNGS and CTM; extra detection (red): pathogens detected exclusively by either mNGS or CTM, with pathogenicity not ruled out; and false-positive detection (blue): microorganisms detected by either mNGS or CTM but clinically diagnosed as non-causative pathogens. CMV: cytomegalovirus; EBV: Epstein-Barr virus; HSV: herpes simplex virus; HHV: human herpes virus.

Among the 55 lung cancer patients diagnosed with PI, 63 episodes of infection were documented. The positive detection rates of mNGS and traditional culture were 93.65% (59/63) and 12.70% (8/63), respectively. The positive rate of mNGS was significantly higher than that of traditional culture (*p* < 0.0001). mNGS and conventional pathogen detection methods identified 41 distinct pathogens, including 14 G− bacteria, 7 gram-positive (G+) bacteria, 11 fungi, 8 viruses, and *M. tuberculosis* complex (MTBC) (Figure 1). Fungi and viruses were the predominant pathogens, identified in 33 and 32 cases, respectively. G− bacteria were found in 27 cases, while G+ bacteria were detected in 19 cases. MTBC was present in three patients.

The most prevalent G− bacteria were *Acinetobacter baumannii* (*n* = 8) and *Pseudomonas aeruginosa* (*n* = 6), followed by *Stenotrophomonas maltophilia* (*n* = 5), *Haemophilus influenza* (*n* = 4), and *Klebsiella pneumonia* (*n* = 4). For G+ bacteria, *Streptococcus pneumoniae* (*n* = 8) and *Enterococcus faecium* (*n* = 4) were the most frequently detected. The pathogenicity of the majority of detected bacteria could not be ruled out, except for two cases of *Nocardia farcinica* and one case of *Bacteroides heparinolyticus*. The predominant fungi identified were *Candida albicans* (*n* = 11) and *Pneumocystis jirovecii* (*n* = 9), although not all were confirmed as causative pathogens. Among viruses, cytomegalovirus (CMV) (*n* = 17) and Epstein-Barr virus (EBV) (*n* = 13) were the most frequently detected, despite their rare association with pathogenicity in immunocompetent hosts. Herpes simplex virus 1 (HSV-1, *n* = 7) and human herpes virus 7 (HHV-7, *n* = 3) were also observed, while adenovirus, which is commonly associated with respiratory infections, was rarely identified in this study.

Of the 63 infection episodes, 4 cases presented with typical clinical symptoms of bacterial infection and responded well to broad-spectrum antibiotic therapy. However, both mNGS and conventional pathogen detection methods failed to identify any causative agents in these cases, suggesting the potential limitations of current diagnostic approaches in capturing all relevant pathogens. Despite these limitations, the overall pathogen spectrum identified in this study provides a comprehensive and reasonably accurate representation of the infectious landscape in lung cancer patients with PI.

### 3.3 Co-detection of the pathogens

**Figure 2:**
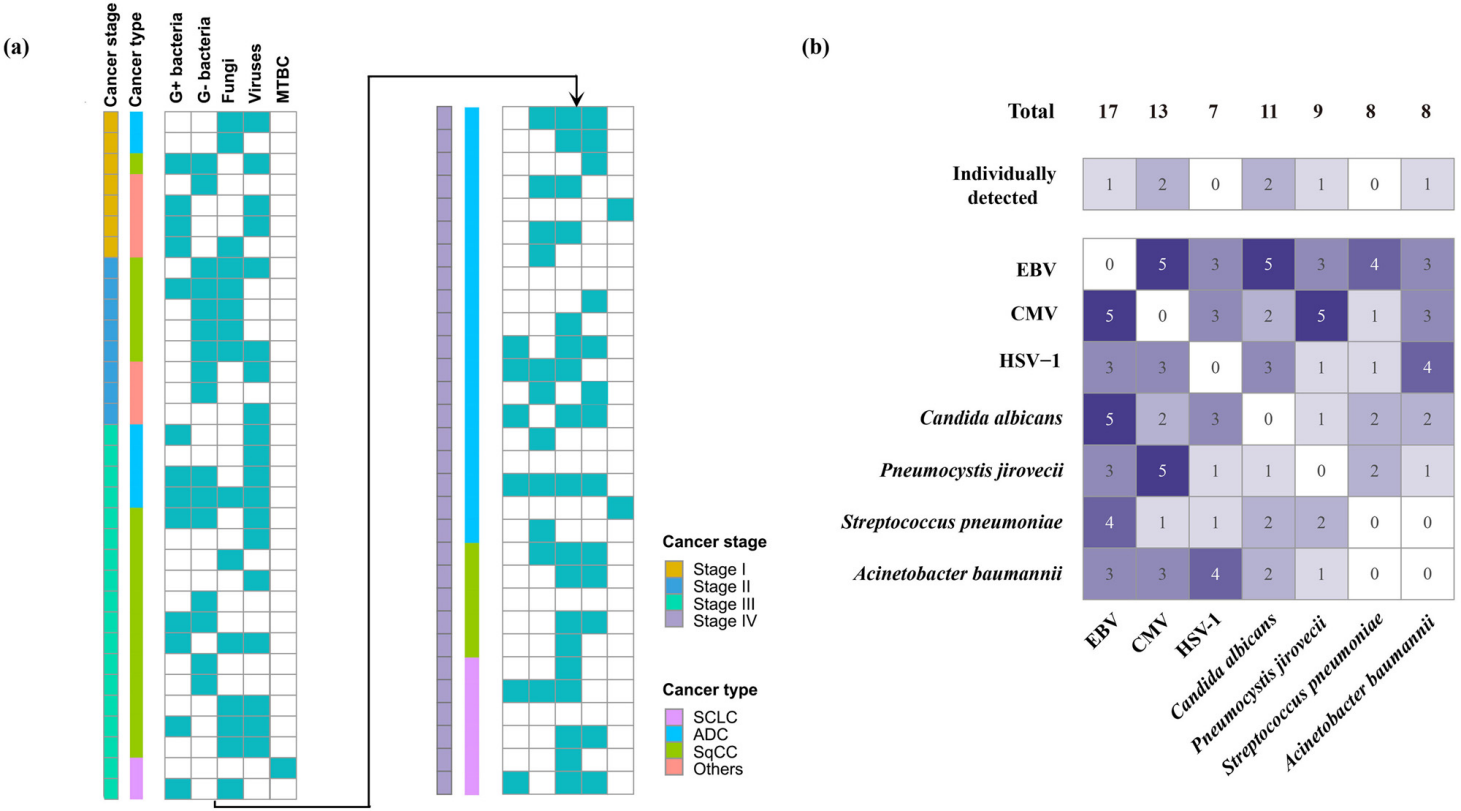
Infection patterns in PI across different cancer types and stages. (a) Infection patterns in 63 infection episodes, with bars color-coded to represent the cancer stage (I–IV) and histological type (NSCLC or SCLC) of each case. Multiple pathogens were detected in 61.02% of infection episodes, with fungi and viruses being the most common co-detected combination. (b) Coexistence patterns of predominant pathogens identified in the cohort, illustrating the complexity of polymicrobial infections in lung cancer patients. *C. albicans* was frequently co-detected with EBV, and *P. jirovecii* was often found alongside CMV. MTBC: *M. tuberculosis* complex; CMV: cytomegalovirus; EBV: Epstein-Barr virus; HSV: herpes simplex virus.

Among the total infection episodes in our study, pathogens were detected in 59 cases using either mNGS or CTM. Of these, only 23 (38.98%) showed the detection of a solitary pathogen, which included 8 G− bacteria, 6 fungi, 5 viruses, and 3 MTBC. In contrast, the remaining 36 infection episodes (61.02%) were found to have multiple pathogens. Among these cases, 19 involved two types of pathogens, with the most common co-detection being fungi and viruses (*n* = 7), followed by G− bacteria and fungi (*n* = 4). In 15 cases, 3 types of pathogens were identified, with the most frequent combination being G+ bacteria, fungi, and viruses (*n* = 5). Additionally, two cases even showed the presence of a mix of G+ bacteria, G− bacteria, fungi, and viruses. Notably, fungi and viruses were co-detected in 18 instances (28.57%), suggesting a potential association between these 2 pathogen types in lung cancer patients with PI (Figure 2a). These observations were consistent across patients with different types and stages of lung cancer, suggesting that the presence of multiple pathogens may not be specific to a particular type or stage of the disease.

The predominant pathogens identified in this cohort, including EBV, CMV, *C. albicans*, *P. jirovecii*, *S. pneumonia*, *A. baumannii*, and HSV-1, were rarely detected individually. Low-pathogenic viruses, such as EBV, CMV, and HSV-1 frequently coexisted with each other and were commonly detected alongside bacteria or fungi. Among the 11 cases of *C. albicans*, 5 exhibited concurrent presence with EBV. Similarly, more than half of the *P. jirovecii* cases were co-detected with CMV. In terms of bacteria, *S. pneumoniae* was more frequently co-detected with EBV and fungi, while *A. baumannii* showed a higher coexistence with the three viruses (Figure 2b).

### 3.4 Differences in the pathogens identified between patients with small-cell lung carcinoma (SCLC) and non-small-cell lung carcinoma (NSCLC)

**Figure 3:**
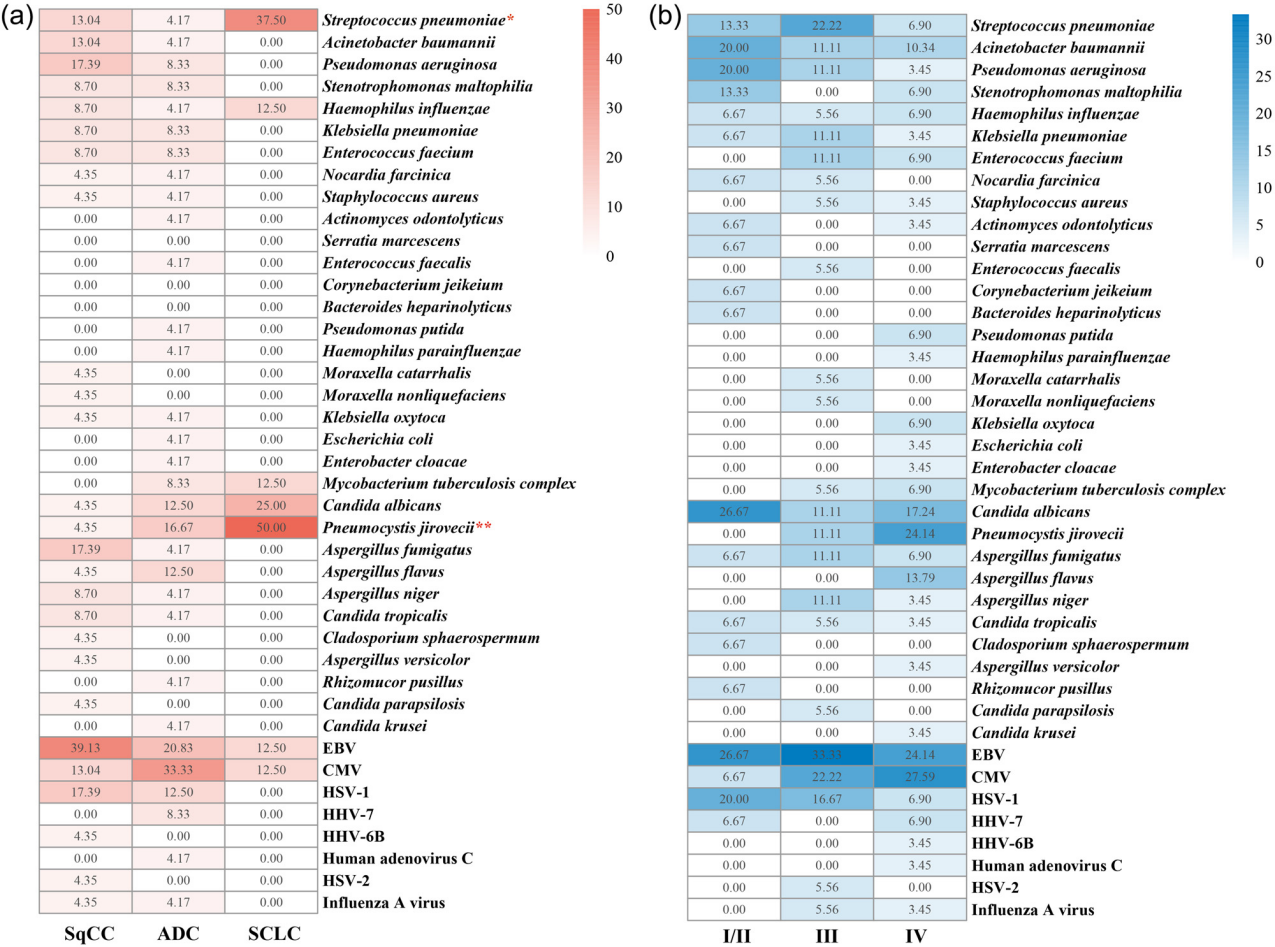
Pathogen distribution across lung cancer stages and histological types. (a) Proportions of pathogens detected in different histological types of lung cancer, calculated as the number of cases positive for a specific pathogen divided by the total number of patients within each cancer type. (b) Proportions of pathogens detected across different cancer stages, calculated as the number of cases positive for a specific pathogen divided by the total number of patients within each stage group. Notable trends include the increased detection of CMV in late-stage patients and the exclusive presence of *P. jirovecii* and *A. flavus* in this group. Statistical significance was determined using the *χ*² test (*p < 0.05; **p < 0.01).

The detected pathogens were further compared between patients with SCLC and those with NSCLC, which included SqCC and ADC. Significant differences in the prevalence of certain pathogens were observed among these groups (Figure 3a).

In SCLC patients, *S. pneumoniae* (*p* = 0.039) and *P. jirovecii* (*p* = 0.009) were significantly more prevalent compared to NSCLC patients, with these two pathogens being most frequently observed in the SCLC group. *H. influenza* and *C. albicans* were also more commonly found in SCLC patients, although these differences did not reach statistical significance. Given the limited sample size of SCLC patients in this study, the results may be subject to bias, necessitating further research to validate and extend these findings.

For patients with SqCC, the predominant pathogens identified were EBV (39.13%), *P. aeruginosa* (17.39%), *Aspergillus fumigatus* (17.39%), and HSV-1 (17.39%). EBV and HSV-1 were also commonly found in ADC patients, while *P. aeruginosa* and *A. fumigatus* were less frequently detected in other groups. In ADC patients, the main detected potential pathogens were CMV (33.33%), EBV (20.83%), and *P. jirovecii* (16.67%). A broader spectrum of bacteria was detected in this group compared to SqCC patients, although the frequency of occurrence for each bacterium was relatively low.

### 3.5 Characteristics of infection in patients with early-stage and late-stage lung cancer

We further investigated the prevalence of different pathogens in patients with early-stage and late-stage lung cancer. Although no statistically significant differences were observed among patients with stages I/II, III, and IV, some notable discrepancies in the occurrence of certain pathogens were identified (Figure 3b).

In early-stage lung cancer patients, EBV and *C. albicans* were the most identified pathogens, with detection rates comparable to those in late-stage patients. *P. aeruginosa* and HSV-1, which also showed high detection rates in early-stage patients, were less frequently detected in stage IV patients. Conversely, CMV was rarely identified in early-stage patients, but its occurrence increased as the disease advanced. Meanwhile, *P. jirovecii* and *Aspergillus flavus* were exclusively found in late-stage patients. Additionally, a greater variety of viruses were detected in late-stage patients compared to early-stage patients, particularly in stage IV. Further investigation is needed to explore the clinical significance of these observations and their potential impact on patient outcomes.

### 3.6 Risk factors associated with PI in patients with lung cancer

**Figure A1:**
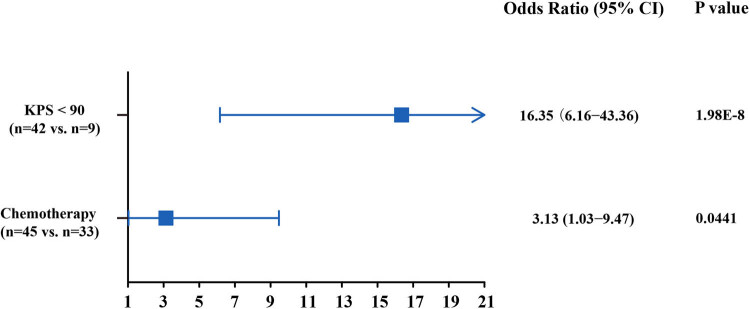
Multivariate analysis of factors associated with pulmonary infections in lung cancer patients.

We also analyzed the underlying risk factors for PI in patients with lung cancer. Univariate analysis revealed that a Karnofsky Performance Status (KPS) score <90 [17], a history of COPD, and advanced-stage cancer were significantly associated with an increased risk of PI. Among the treatments and management measures administered to the patients, invasive examination, chemotherapy, and immunotherapy were significantly correlated with an increased risk of PI (Table 2). Multivariate analysis further identified two independent risk factors significantly associated with PI in lung cancer patients: a KPS score of <90 and chemotherapy treatment (Figure A1).

**Table 2:** Univariate analysis of risk factors for PI in lung cancer patients

	Patients with PI (*n* = 55)	Controls (*n* = 59)	OR	95% CI	*P* value
**Baseline characteristics**					
Age	64	63	0.96	0.93–1.01	0.0924
COPD	16 (29.09%)	8 (13.56%)	0.38	0.15–0.98	0.0463
**Clinical course**					
KPS < 90	42 (76.36%)	9 (15.25%)	0.06	0.02–0.14	<0.0001
Invasive examination	54 (98.18%)	50 (84.75%)	0.10	0.01–0.84	0.0339
**Treatments received for lung cancer**					
Surgery	8 (14.81%)	11 (18.64%)	1.35	0.5–3.64	0.5582
Radiotherapy	14 (25.93%)	14 (23.73%)	0.91	0.39–2.14	0.8307
Chemotherapy	45 (81.82%)	33 (55.93%)	0.28	0.12–0.66	0.0038
Immunotherapy	24 (43.64%)	12 (20.34%)	0.33	0.14–0.75	0.0087
Targeted therapy	9 (16.36%)	4 (6.78%)	0.37	0.11–1.29	0.1181
NSCLC	42 (76.36%)	49 (83.05%)	1.22	0.43–3.5	0.7054
Late stage	42 (76.36%)	54 (91.53%)	3.34	1.1–10.12	0.0327

PI: pulmonary infection; COPD: chronic obstructive pulmonary disease; CI: confidence interval; KPS: Karnofsky performance status; NSCLC: non-small-cell lung carcinoma.

## 4 Discussion

In this study, we comprehensively analyzed the clinical characteristics, pathogen spectrum, and risk factors associated with PI in 55 lung cancer patients. Our findings highlighted the complexity of PI in the lung cancer patient population, emphasizing the diverse microbial landscape and the multifactorial nature of infection risk.

The demographic and clinical characteristics of the study population revealed that lung cancer patients with PI were more likely to have underlying COPD, a finding consistent with previous studies that have identified COPD as a significant risk factor for respiratory infections in cancer patients [18]. The high prevalence of advanced-stage cancer (stages III and IV) in our cohort further underscores the vulnerability of these patients to infections, likely due to immunosuppression caused by both the disease and its treatment.

The pathogen landscape in lung cancer patients with PI was notably diverse, with fungi and viruses being the most frequently identified pathogens. This finding aligns with prior research indicating that immunocompromised patients, particularly those undergoing chemotherapy or immunotherapy, are at increased risk of opportunistic infections caused by fungi and viruses [19]. The predominance of *C. albicans*, *Aspergillus*, and *P. jirovecii* among fungi, as well as CMV and EBV among viruses, highlights the importance of considering these pathogens in the diagnostic and therapeutic management of lung cancer patients with PI [20,21,22,23].

Interestingly, the co-detection of multiple pathogens was a common occurrence, with fungi and viruses frequently coexisting. This observation suggests that the interplay between different types of pathogens may contribute to the complexity of PI in lung cancer patients. For instance, mixed infections may arise from conditions such as partial airway obstruction and immunological dysregulation [24,25]. Respiratory viruses, for instance, can facilitate bacterial invasion into the lower airways, leading to more severe clinical outcomes [25,26,27]. Similarly, viral and fungal co-infections have been associated with increased mortality in patients with PI, involving intricate interactions between host immune defenses and microbial virulence [28]. Further research is needed to explore the clinical implications of these co-infections and their impact on patient outcomes. The use of advanced diagnostic tools, such as mNGS, alongside CTM, may play a critical role in identifying the causative agents and guiding targeted therapy.

The study also revealed significant differences in pathogen prevalence between SCLC and NSCLC patients. SCLC patients exhibited a higher prevalence of *S. pneumoniae* and *P. jirovecii*. In contrast, EBV was more frequently observed in NSCLC patients. This may reflect differences in immune status or treatment regimens between these two groups [29,30,31]. These findings suggest that the type of lung cancer may influence the risk of specific infections, warranting tailored diagnostic and preventive strategies.

Regarding cancer staging, although no statistically significant differences in pathogen prevalence were observed between early-stage and late-stage patients, certain trends were notable. CMV demonstrated a particularly interesting pattern, being rarely detected in early-stage patients but showing increased prevalence with disease progression. This trend likely reflects the progressive immunosuppression characteristic of advanced cancer stages. As a virus possessing both immunosuppressive and inflammatory properties, CMV may facilitate tumor immune evasion through its ability to impair the cytotoxic functions of natural killer cells and T lymphocytes [32]. Furthermore, CMV can promote existing inflammatory pathways, leading to its own propagation within the body and further compromising the host’s health [33,34]. Notably, emerging evidence suggests that CMV infection may play a significant role in the development of immune checkpoint inhibitor-associated pneumonitis in lung cancer patients [35]. Conversely, pathogens such as *P. aeruginosa* and HSV-1 were more common in early-stage patients, possibly due to differences in treatment modalities or immune responses at different stages of the disease.

The identification of risk factors for PI in lung cancer patients constitutes a critical component of this study, particularly given the evolving treatment landscape and associated infection risks. Univariate analysis revealed that a history of COPD, advanced-stage cancer, invasive examinations, chemotherapy, and immunotherapy were significantly associated with an increased risk of PI. Subsequent multivariate analysis refined these findings, identifying a KPS score of <90 and chemotherapy as independent risk factors for PI. These findings align with established literature demonstrating the dual impact of chemotherapy-induced neutropenia and cancer-related immune deficiencies on infectious complications [5]. The strong association between chemotherapy and PI risk reflects well-documented treatment-related immunosuppression, while the correlation with poorer performance status (KPS < 90) likely reflects both disease burden and compromised host defenses. Notably, while our study confirms these established risk factors, it also highlights the need for continued vigilance in the era of novel therapies. The emergence of immunotherapy and targeted therapies has fundamentally transformed lung cancer treatment paradigms, yet our understanding of their infection risk profiles remains incomplete [36,37,38]. This evolving therapeutic landscape underscores the importance of comprehensive infection risk management strategies, including judicious use of prophylactic antibiotics and rigorous monitoring of immune status throughout treatment. Such measures are particularly crucial as treatment regimens become increasingly complex and patient populations more diverse in their risk profiles.

mNGS could play a key role in this context due to its ability to rapidly and comprehensively detect a wide range of pathogens, including bacteria, fungi, and viruses. Proactive mNGS-based surveillance in high-risk patients could enable early detection of pathogens, even before clinical symptoms manifest. This would allow for the timely initiation of targeted therapy, potentially reducing the severity and complications of PI. Given the high prevalence of mixed infections in our study, mNGS is particularly valuable for identifying co-detected pathogens that might be missed by CTM. While mNGS is a powerful tool, its cost and resource requirements may limit its widespread use. Therefore, proactive screening could initially be targeted to the highest-risk patients, such as those with advanced-stage cancer, poor performance status, or those receiving intensive chemotherapy. Further cost-effectiveness studies are needed to evaluate the feasibility of implementing mNGS-based surveillance in routine clinical practice.

While this study provides valuable insights into the clinical and microbiological characteristics of PI in lung cancer patients, several limitations should be acknowledged. First, the single-center design may limit the generalizability of our findings. Future studies should adopt a multi-center design, enrolling patients from diverse geographic and clinical settings. This would not only enhance the external validity of the findings but also allow for the exploration of regional variations in pathogen distribution and infection risk. Second, our study focused on DNA-based mNGS, which is necessary for detecting RNA viruses. This limitation may have led to an underestimation of the role of RNA viruses in PI among lung cancer patients. Third, the relatively small sample size, particularly for SCLC patients and early-stage lung cancer patients, may limit the statistical power and generalizability of our findings. Larger, prospective studies are needed to validate these observations and explore potential associations that may not have reached significance in our cohort. Fourth, while the combined use of mNGS and conventional diagnostic methods offers a comprehensive approach to pathogen detection, these techniques have inherent limitations. For example, mNGS may detect environmental contaminants or commensal organisms, complicating the interpretation of results. Improved methods for differentiating infection from colonization are needed to enhance diagnostic accuracy. Finally, our study did not explore the impact of specific treatment regimens (e.g., different chemotherapy agents or immunotherapy protocols) on infection risk. Given the growing use of targeted therapies and immunotherapies in lung cancer, future studies should examine how these treatments influence the risk and spectrum of PI. Addressing these limitations in future research will provide a more comprehensive understanding of PI in lung cancer patients and improve clinical management strategies.

## 5 Conclusion

In conclusion, this study highlights the diverse pathogen spectrum and multifactorial risk factors associated with PI in lung cancer patients. The findings underscore the importance of considering both the type and stage of lung cancer, as well as the patient’s treatment history and performance status, when assessing infection risk. The high prevalence of co-infections involving fungi and viruses suggests that a multifaceted approach to diagnosis and treatment may be necessary to improve outcomes in this vulnerable population. Further research is needed to explore the clinical implications of these findings and to develop targeted strategies for the prevention and management of PI in lung cancer patients.
